# Construct Validity of the Chinese Version of the Psycho-Educational Profile-3rd Edition (CPEP-3)

**DOI:** 10.1007/s10803-014-2143-5

**Published:** 2014-05-17

**Authors:** Daniel Tan Lei Shek, Lu Yu

**Affiliations:** Department of Applied Social Sciences, The Hong Kong Polytechnic University, Hung Hom, Kowloon, Hong Kong

**Keywords:** Assessment, Autism spectrum disorder, Chinese, Construct validity, Psycho-Educational Profile-3rd edition, Psychometric properties

## Abstract

Objective behavioral assessment of autism spectrum disorder (ASD) in early childhood is essential for guiding appropriate treatment and intervention. In contrast to Western societies, validated measures of ASD are very limited in different Chinese contexts. The present study attempted to examine the construct validity of the Chinese version of Psycho-Educational Profile-3rd edition (CPEP-3). The CPEP-3 was administered to a sample of 455 children with ASD and a comparison group of 281 children without ASD. As predicted, older children scored significantly higher than younger children on different subtests of CPEP-3, and there was no gender difference within the autistic group. The construct validity of the CPEP-3 was further supported by the high internal consistency of each subtest as well as the moderate to large correlation coefficients among subtests. In line with the theoretical model, confirmatory factor analysis showed the three-factor model of the Performance test fitted well. In conjunction with the data reported previously, the present findings provided sound evidence for the construct validity of CPEP-3.

## Introduction

Despite the fact that much attention has been paid to children with autistic spectrum disorders (ASD) globally, relatively few studies have been conducted in different Chinese contexts. In a recent review on the prevalence of autism in mainland China, Hong Kong and Taiwan, Sun et al. (2013) criticized that available studies in different Chinese contexts “have methodological weaknesses” and “the results lack comparability with those from developed countries” (p. 1). Their meta-analytic findings also suggested a potential under-diagnosis and under-detection of ASD in Chinese communities and argued for the need to use more advanced methods for research of ASD (Sun et al. 2013). With specific reference to Hong Kong, it was not until the early 1990s that public awareness of autism began to increase. More and more parents started to call for government attention and resources to help their autistic children. As a result, local services aiming at children with ASD have been gradually grown and the cultural acceptance of ASD has been improved in Hong Kong society (Wong and Hui 2008a).

One milestone in the development of service for children with ASD in Hong Kong was the introduction of the renowned Treatment and Education of Autistic and related Communication handicapped CHildren (TEACCH) program by the Heep Hong Society in 1993. The TEACCH program adopts structured teaching strategies to facilitate learning and skills-building in children with ASD and to reduce their disruptive behavior (Schopler 1997). To accurately assess the development of children with pervasive developmental disorders and design individualized training plans, the TEACCH division in the North Carolina University (Schopler et al. 1990) developed a revised instrument called the Psychoeducational Profile-Revised (PEP-R). The PEP-R provides a useful framework for researchers and practitioners to formulate suitable education plan and ongoing evaluation of autistic children. The Heep Hong Society also translated this instrument into Chinese and conducted a validation study to examine the psychometric properties of the Chinese version of the PEP-R (CPEP-R; Shek et al. 2005).

Based on a sample of 63 preschool children with symptoms of ASD in Hong Kong, Shek et al. (2005) found that different domains of CPEP-R had very good reliability in terms of internal consistency (Cronbach’s alpha ranged from 0.74 to 0.98), inter-rater reliability (intra-class correlation coefficients ranged from 0.84 to 0.87) and test–retest reliability (Pearson correlation coefficients ranged from 0.76 to 0.92). It was also reported that the CPEP-R scores were significantly correlated with the Merrill-Palmer Scale of Mental Tests (Stutsman 1948) and the Hong Kong Based Adaptive Behavior Scale (Kwok et al. 1989). These observations clearly provided support for the concurrent validity of the instrument. In the past years, the Chinese version of PEP-R has been widely used to assess the cognitive ability, social adaptive functioning, and developmental abilities in children with ASD in Hong Kong. Besides, it has been used by practitioners as an outcome measure when evaluating the effectiveness of educational programs for children with ASD.

In 2005, Schopler et al. further revised the PEP-R into a more comprehensive version—the Psycho-Educational Profile-3rd edition (PEP-3) for children with ASD whose developmental age is from 6 months to 7 years. Compared to the PEP-R, the PEP-3 has more concrete and interesting materials, limited verbal demands, and untimed administration process. Besides, the language items were separated from the general items (Chen et al. 2011; Schopler et al. 2005). According to Schopler et al. (2005), the PEP-3 is a reliable and valid instrument which has the potential to assess and monitor the development of children with ASD in a more accurate and comprehensive way. Based on a sample of children with developmental disorders in the United States, Schopler et al. (2005) reported good internal consistency, test–retest reliability, and inter-rater reliability for the PEP-3. The high correlations between PEP-3 and other measures assessing similar developmental constructs were also reported, providing support for the validity of the instrument. However, except for the findings based on the validation study reported in the PEP-3 manual, there are few publications on the psychometric properties of the PEP-3.

Among the limited studies, Fulton and D’Entremont (2013) examined the ability of the PEP-3 in estimating cognitive and language skills of 136 children with ASD (aged 20–75 months) in Canada. Positive correlations were found between the PEP-3 cognitive and language measures and similar measures including the Child Development Inventory (Ireton 1992), the Merrill-Palmer Revised Developmental Scale (Roid and Sampers 2004), and the Vineland Adaptive Behavior Scale-2 (Sparrow et al. 2005). Significant differences in performances on PEP-3 cognitive and language measures were detected among three diagnostic groups of children with ASD, Asperger’s disorder, or pervasive developmental disorders. These findings provided support for the psychometric properties of the subtests of PEP-3 as an assessment tool measuring cognitive and language skills in children. Nonetheless, the reliability and validity of the subtests focusing on maladaptive behaviors (e.g., social reciprocal, affective expression, characteristic motor behavior, and characteristic verbal behavior) were not investigated in Fulton and D’Entremont’s (2013) study.

In Taiwan, a group of researchers translated the PEP-3 into Mandarin Chinese and administered it in a sample of 63 children with ASD. While the reliability and validity of the Caregiver Report of the PEP-3 were supported (Fu et al. 2010, 2012), psychometric properties of the major part of PEP-3 (i.e., the Performance test) remain largely unknown probably because of the small sample of the study. Chen et al. (2011) reported good sensitivity of the Performance test, i.e., the ability of the measure to detect change over time and in response to an intervention (Guyatt et al. 1992), which is the only available psychometric paper on the Performance test in Chinese children. As such, the psychometric properties of the PEP-3 for the assessment of Chinese children with ASD need to be further demonstrated.

Against this background, researchers in Hong Kong translated the PEP-3 into Cantonese Chinese and conducted a validation study based on a large sample of autistic children and a comparison group of normal children in Hong Kong. Shek and Yu (2013) reported that the PEP-3 performance test showed good psychometric properties in terms of internal consistency, test–retest reliability, inter-rater reliability, content validity, and concurrent validity. While these results lent support for the reliable and valid use of CPEP-3 in Chinese population, the construct validity of the instrument was not examined. As such, the present study attempted to investigate the construct validity of the CPEP-3.

Construct validity refers to the extent to which an instrument measures the construct it claims to be measuring or the degree to which the underlying traits of the test can be identified (Anastasi and Urbina 1997). If a test lacks construct validity, results obtained by this measure will not be interpretable. Therefore, construct validity should be considered at the heart of any study when researchers use an instrument to measure a construct that is not directly observable (Cronbach and Meehl 1955). To accumulate sound evidence for the psychometric properties of a measure, construct validity must be established. According to Singleton and Straits (1999), “evidence of construct validity consists of any empirical data that support the claim that a given operational definition measures a certain concept.” (p. 124) Four common types of evidence have been highlighted to establish construct validity, which include a) correlations with related variables (i.e., convergent validity); b) consistency across measures and methods of measurement (i.e., external validity); c) correlations with unrelated variables (i.e., discriminant validity); and d) differences between contrasted groups (i.e., contrasted groups validity). Some researchers also suggest factorial validity (i.e., the extent to which the data conform to the hypothesized dimensions of the measure) as a form of construct validity (Dooley 1990). The present study aimed to examine the construct validity of the CPEP-3 in terms of three aspects: (a) correlations with related variables and unrelated variables; (b) differences between contrasted groups, and (c) factorial validity.

Specifically, six hypotheses regarding four types of validity evidence were proposed and tested. The first two hypotheses were posited to provide evidence for the correlations between CPEP-3 subtests and related variables as well as unrelated variables. Primarily, different subtests were assumed to have different relationships with participants’ age. As seven subtests were designed to measure developmental skills (i.e., cognitive verbal/preverbal, expressive language, receptive language, fine motor, gross motor, visual-motor imitation, and personal self-care), it was hypothesized that the scores would be correlated with participants’ age. In other words, older children were assumed to have higher scores on these subtests than did younger children (Hypothesis 1a). On the other hand, six subtests measuring maladaptive behaviors, including affective expression, social reciprocity, characteristic motor behaviors, characteristic verbal behaviors, problem behavior, and adaptive behaviors, should be weakly correlated with age (Hypothesis 1b). Schumm et al. (1986) suggested that a relevant correlation coefficient with a magnitude of at least 0.4 would be needed to establish convergent validity whereas a related correlation coefficient of 0.3 or less would provide evidence for the discriminant validity of the test. These criteria were adopted in the present study for the first two hypotheses testing.

In addition to age, it was hypothesized that gender would not be related to CPEP-3 subtests (Hypothesis 1c) in the sample of autistic children. It should be noted that although some researchers reported that girls appeared to have more severe autism than did boys, the findings are inconsistent and no strong evidence suggests that autistic boys tend to be higher functioning than autistic girls. Besides we advanced this hypothesis based on the hypothesis described in the PEP-3 manual.

Second, to examine differences between contrasted groups, one hypothesis was proposed. Since the CPEP-3 was devised to assess the characteristics of children with autistic disorders, it was hypothesized that autistic children would score lower than typically developing children (Hypothesis 2) on the ten Performance subtests.

Third, to test the factorial validity of CPEP-3, another two hypotheses were examined. Because different subtests of CPEP-3 measure different aspects of development and behaviors, they were expected to be moderately correlated with each other (Hypothesis 3). Besides, it was theoretically suggested that the ten Performance subtests would contribute to three domains (communication, motor skills, and maladaptive behavior), which reflect autistic children’s overall development in communication functions, motor skills, and presence of maladaptive behaviors, respectively. Particularly, cognitive verbal/preverbal, expressive language, receptive language would load on the factor “communication”; fine motor, gross motor, visual-motor imitation would load on the factor of motor, and affective expression, social reciprocity, characteristic motor behaviors, and characteristic verbal behaviors would contribute to the factor of maladaptive behavior. The factor structure of the three domains relating to their respective subtests should be supported by confirmatory factor analysis (Hypothesis 4).

In addition, although internal consistency is typically employed as an index of reliability, there are views considering internal consistency, a measure of the inter-relatedness of the items within a test, as an indicator to confirm whether or not a group of items are measuring the same construct/concept (Cortina 1993). Some researchers (Tavakol and Dennick 2011) proposed that internal consistency (e.g., Cronbach’s alpha) adds “validity and accuracy to the interpretation of their data” (p. 55). In the original test manual, internal consistency is regarded as an additional evidence of construct validity. Therefore, we also examined internal consistency for each subtest to provide further evidence for the construct of CPEP-3. It was expected that the construct validity of the CPEP-3 could be established with evidence obtained by testing the above hypotheses.

## Methods

### Participants

Data were collected from 455 children who were diagnosed as having autism or other pervasive developmental disorders (PDDs) in 25 service units in the Heep Hong Society including special child care centers, early education and training centers, and parent resource centers. Another sample of 281 children without developmental problems was also selected as the “normal sample” from 13 local kindergartens matched for age with the autistic sample for comparison. The diagnoses of the autistic sample were made based on ICD-10/DSM-IV by consultant psychiatrists and endorsed by a multidisciplinary team consisting of clinical psychologists, special educators, and other helping professionals. Several subgroups of participants were randomly selected for the analyses of test–retest reliability, inter-rater reliability, and criterion-prediction validity (Shek and Yu 2013). Specifically, based on a subsample of 42 autistic children, correlation coefficient for each subtest at two time points over a period of 6 weeks to 3 months ranged from 0.84 to 0.99, suggesting good test–retest reliability. Inter-rater reliability indicated by polychoric correlation coefficients for each pair of items rated by two experienced examiners ranged from 0.34 to 0.78 (n = 46). Criterion-prediction validity was also found to be good. Details of the study were reported elsewhere (Shek and Yu 2013).

Participants’ age ranges from 2.0 to 7.9 years. Table 1 summarizes the characteristics of the two samples in terms of age and gender. For the autistic sample, the ratio of boys to girls is 6:1, reflecting the fact that boys had higher risk of autistic disorders than did girls. While this figure was higher than the related ratio in the American normative sample (4:1); it was highly similar to the findings of a large epidemiological study of autistic spectrum disorder in which the male to female ratio was found to be 6.58:1 in Hong Kong children (Wong and Hui 2008b).

**Table 1:** Demographic characteristic of the samples

Age group	2	3	4	5	6	7	Total
Age range in years	2.0–2.9	3.0–3.9	4.0–4.9	5.0–5.9	6.0–6.9	7.0–7.9	
Autistic sample							
No. of participants	32	79	140	161	37	6	455
Percentage	7.0	17.4	30.8	35.4	8.1	1.3	100
No. of girls	6	11	22	22	5	0	66
No. of boys	26	68	118	139	32	6	389
Normal sample							
No. of participants	67	60	60	62	30	2	281
Percentage	23.8	21.4	21.4	22.1	10.7	0.7	100
No. of girls	34	37	36	30	14	1	152
No. of boys	33	23	24	32	16	1	129

### Procedure

The present study was conducted at the Heep Hong Society, which has over 30 service units in different parts of Hong Kong. Children with a suspected diagnosis of ASD are referred to the service centers of the Heep Hong Society by different hospitals and schools in various areas of Hong Kong. Children with problem behaviors and developmental delay are also brought to the centers by their caregivers or teachers. For the present study, a group of professionals including speech therapists, occupational therapists, developmental psychologists, and preschool teachers administered the Performance tests and rated the participants. All raters had experience working with and testing young children. Before the formal launch of the validation study, the raters worked together to clarify and get familiar with general testing, scoring and interpreting procedures of the CPEP-3 to ensure consistency in the test administration.

For the Caregiver Report of the CPEP-3, the researchers explained the procedure and purpose of the test and gave clear instructions on how to fill in the report to parents. The Caregiver Report was then completed by either the mother or father of the participating child. During the process of completing the report, one researcher was present and gave explanations when parents had any doubts regarding the questions. The researchers who administered the Performance Test were blind to the scores of Caregiver Report, and vice versa. For parents of the normal sample, they only completed the personal self-care (PSC) subtest in the Caregiver Report as the items for problem behavior (PB) and adaptive behavior (AB) are not applicable to normal children.

### Instruments

#### The Chinese Version of Psycho-Educational Profile-3rd Edition (CPEP-3)

The PEP-3 developed by Schopler et al. (2005) has two major parts: Performance and Caregiver Report. The 172-item Performance section is composed of 10 subtests. Three subtests measure communication ability including cognitive verbal/preverbal (34 items), expressive language (25 items), and receptive language (19 items). Another three subtests measure motor ability: fine motor (20 items), gross motor (15 items), and visual-motor imitation (10 items). These six subtests focus on the child’s development level. The remaining four subtests measure maladaptive behaviors, including affective expression (11 items), social reciprocity (12 items), characteristic motor behaviors (15 items), and characteristic verbal behaviors (11 items). The Caregiver Report consists of 38 items which are combined into three subtests: problem behavior (10 items), personal self-care (13 items), and adaptive behavior (15 items).

Authorized by the PEP-3 developers, the Heep Hong Society organized a working group to translate the PEP-3 items into Chinese, with the first author as the chairman of the specialist working group. The translated draft was then reviewed and modified by the group after discussion. Compared with the English version of the PEP-3, major changes in the CPEP-3 were in the areas of language and use of stimuli. Adaptation and modifications were made after taking into account the cultural and language factors. Chinese words were used to replace the English ones in the items for letter matching, naming and sorting, and a few more culturally suitable pictures were used to replace the original ones. The translated test is administered in Cantonese. The scoring of items has been quantified as 0, 1, and 2, with “Pass” = 2, “Emerge” = 1, and “Fail” = 0. In the present study, raw score obtained from each item was used for all data analyses.

### Data Analysis

First, correlation coefficients between participants’ raw scores on CPEP-3 subtests and age were computed to provide evidence for the first two types of construct validity: correlations with related and unrelated variables (Hypotheses 1a and 1b). To examine the relationship between CPEP-3 and gender, a MANOVA was conducted with participants’ scores on different subtests served as dependent variables and gender as independent variable (Hypothesis 1c). Second, to investigate differences between contrasted groups, another MANOVA was conducted to compare typically developing children and autistic children in their CPEP-3 Performance subtest scores (Hypothesis 2).

Third, factorial validity was tested by computing the correlation coefficients among different CPEP-3 subtest scores (Hypothesis 3) and performing a confirmatory factor analysis based on the autistic sample of children (Hypothesis 4). The hypothesized factorial model consists of three latent variables (i.e., the three CPEP-3 composites) and ten observed variables (i.e., the ten subtests). The three composites (Communication, Motor, and Maladaptive Behavior) were allowed to be correlated because they measure related but different aspects of development and behavior. It is assumed that each subtest has a non-zero loading on its related factor and zero loadings on other factors. Specifically, CVP, EL, and RL load on Communication; FM, GM, and VMI load on Motor, and AE, SR, CMB, and CVB load on Maladaptive Behavior. Using AMOS 17.0, 455 autistic children’s raw scores on the 10 Performance subtests were subject to the CFA Procedure using maximum likelihood method. To evaluate how well the model fits the sample data, five indexes of model fit were calculated, including the comparative fit index (CFI), Tucker and Lewis’s index of fit (TLI), normed fit index (NFI), root mean square error of approximation (RMSEA), and standardized root mean square residual (SRMR). Although the criterion for acceptable model fit varies from study to study, the same rules used by the PEP-3 developers were adopted: (a) CFI, TLI, and NFI values should be equal to or above 0.90 to indicate a satisfactory model fit, with values close to 1 suggesting a very good fit on any of the indexes; (b) for RMSEA, a value of less than 0.08 indicates a reasonable fit and a value of less than 0.05 or less indicates good model fit in relation to the degrees of freedom. In addition, as SRMR was advocated as the most sensitive to structural model misspecification (Hu and Bentler 1995), it was adopted as an extra index in this study. A value of less than 0.08 is generally considered as a good fit.

Lastly, internal consistency of the instrument was examined by calculating the item-total correlation of each subtest using the autistic sample, which would add “validity and accuracy to the interpretation of” (Tavakol and Dennick 2011, p. 55) the data based on the instrument.

## Results

### Correlations with Age and Gender (Hypotheses 1a, 1b and 1c)

Tables 2 and 3 present participants’ mean scores on subtests of CPEP-3 at different age groups and the overall correlation coefficients between CPEP-3 subtest scores and age. Consistent with Hypothesis 1a, scores on the seven developmental subtests (CVP, EL, RL, FM, GM, VMI, and PSC) were significantly correlated with age for both the autistic sample and the normal sample. The correlation coefficients were in the moderate to large range. For the autistic sample, the correlation coefficients ranged 0.45–0.55 (values in italic in Table 2); for the normal sample, the coefficients ranged from 0.44 to 0.53 (values in italic in Table 3). Older children had higher scores than did younger children. These findings suggest that the subtests are sensitive to the developmental nature of the subtests’ contents, which provide support for the construct validity of the CPEP-3.

**Table 2:** Means and standard deviations for CPEP-3 scores at different ages and correlation coefficients with age on the autistic sample (N = 455)

Age	CVP	EL	RL	FM	GM	VMI	AE	SR	CMB	CVB	PB	*PSC*	AB
2	22.81 (11.06)	7.31 (6.74)	12.63 (9.49)	23.97 (5.87)	18.25 (5.76)	8.19 (4.51)	14.78 (4.58)	11.31 (4.72)	20.78 (5.73)	6.50 (6.05)	8.72 (3.20)	11.53 (4.55)	16.38 (5.00)
3	35.38 (16.24)	14.76 (12.20)	19.32 (11.73)	29.49 (7.16)	23.85 (6.06)	11.78 (5.14)	14.62 (4.37)	13.59 (4.34)	21.52 (6.43)	9.61 (6.82)	8.96 (3.30)	14.70 (4.11)	17.89 (5.04)
4	45.74 (15.92)	22.53 (13.66)	25.74 (10.98)	33.74 (5.99)	26.86 (4.40)	15.14 (4.32)	16.17 (4.67)	16.24 (4.88)	24.02 (5.60)	12.69 (6.33)	9.74 (3.98)	17.64 (3.74)	19.16 (5.58)
5	52.06 (16.71)	27.75 (14.86)	28.96 (10.82)	35.39 (5.93)	27.86 (4.07)	15.65 (4.42)	17.22 (4.55)	17.17 (5.26)	24.35 (5.77)	13.52 (6.78)	10.29 (3.75)	19.10 (4.07)	19.78 (5.51)
6	59.38 (8.44)	38.03 (9.36)	34.00 (5.56)	37.16 (2.82)	28.86 (1.40)	17.84 (2.22)	18.59 (2.02)	18.92 (3.44)	25.65 (4.72)	17.38 (3.77)	11.32 (3.37)	20.68 (2.88)	21.38 (5.60)
7	63.17 (2.71)	42.00 (7.38)	35.67 (1.97)	38.50 (1.64)	29.00 (0.00)	19.00 (0.63)	20.17 (2.14)	21.33 (3.27)	28.83 (0.75)	18.67 (2.50)	11.67 (1.75)	21.50 (1.52)	21.67 (4.03)
Correlation coefficients with age^a^	*0.52*	*0.50*	*0.46*	*0.48*	*0.47*	*0.45*	0.26	0.38	0.24	0.36	0.19	*0.55*	0.23

Values in the parentheses are standard deviations

Variables and values in italic are subtests measuring developmental skills which were hypothesized to be strongly correlated with age and the related coefficients; variables and values underlined are subtests measuring maladaptive behaviors supposed to be weakly correlated with age and the related coefficients

*CVP* cognitive verbal/preverbal, *EL* expressive language, *RL* receptive language, *FM* fine motor, *GM* gross motor, *VMI* visual-motor imitation, *AE* affective expression, *SR* social reciprocity, *CMB* characteristic motor behaviors, *CVB* characteristic verbal behaviors, *PB* problem behavior, *PSC* personal self-care, *AB* adaptive behavior

^a^All correlation coefficients are statistically significant

**Table 3:** Means and standard deviations for CPEP-3 scores at different ages and correlation coefficients with age on the normal sample (N = 281)

Age	CVP	EL	RL	FM	GM	VMI	AE	SR	CMB	CVB	PSC
2	42.03 (5.43)	22.72 (6.00)	28.87 (3.63)	31.43 (3.74)	26.39 (2.49)	15.43 (2.33)	20.15 (1.55)	20.45 (2.18)	28.79 (1.23)	19.67 (2.65)	15.69 (3.56)
3	51.18 (5.32)	30.20 (5.33)	32.75 (3.27)	36.70 (2.57)	28.78 (1.30)	17.50 (2.45)	20.50 (1.84)	22.10 (1.98)	29.37 (1.18)	20.23 (1.92)	18.80 (2.60)
4	62.00 (4.07)	37.68 (4.73)	36.65 (1.92)	38.68 (1.27)	29.15 (0.82)	18.93 (1.19)	21.30 (1.64)	22.52 (1.92)	29.63 (1.12)	21.13 (1.47)	21.18 (2.67)
5	65.95 (1.81)	42.94 (3.52)	37.55 (0.72)	39.45 (0.67)	29.65 (0.55)	19.55 (0.76)	21.48 (1.20)	23.13 (1.21)	29.76 (0.74)	21.63 (0.81)	23.44 (1.93)
6	66.50 (1.61)	45.17 (3.40)	37.80 (0.48)	39.40 (0.77)	29.70 (0.53)	19.33 (1.21)	21.50 (1.17)	23.00 (1.23)	29.43 (1.87)	21.83 (0.59)	23.47 (2.26)
7	66.50 (2.12)	47.50 (0.71)	37.50 (0.71)	38.50 (0.71)	29.00 (1.41)	19.00 (1.41)	21.00 (1.41)	22.00 (2.83)	29.00 (1.41)	22.00 (0.00)	24.00 (2.83)
Correlation coefficients with age^a,b^	*.50*	*.50*	*.44*	*.45*	*.44*	*.44*	.27	.38	.24	.36	*.53*

Values in the parentheses are standard deviations

Variables and values in italic are subtests measuring developmental skills which were hypothesized to be strongly correlated with age and the related coefficients; variables and values underlined are subtests measuring maladaptive behaviors supposed to be weakly correlated with age and the related coefficients

*CVP* cognitive verbal/preverbal, *EL* expressive language, *RL* receptive language, *FM* fine motor, *GM* gross motor, *VMI* visual-motor imitation, *AE* affective expression, *SR* social reciprocity, *CMB* characteristic motor behaviors, *CVB* characteristic verbal behaviors, *PSC* personal self-care

^a^All correlation coefficients are statistically significant

^b^No data on PB (problem behavior) and AB (adaptive behavior) subtests were collected from the normal sample

For the six behavioral subtests (AE, SR, CMB, CVB, PB, and AB), although their relationships with age were also statistically significant, the correlation coefficients were relatively low (i.e., with low effect size). In the autistic group, correlation coefficients ranged from 0.19 to 0.38 (values underlined in Table 2); in the normal group, the coefficients ranged from 0.24 to 0.38 (values underlined in Table 3). This indicates that maladaptive behaviors measured by these scales were not closely related to age. It should be noted that no data on two maladaptive behavior subtests (PB and AB) were collected for children in the normal group, and the correlation coefficients between these two subtests and age in normal children were unavailable. Based on Schumm et al.’s (1986) criteria, the current findings basically give support to Hypothesis 1b.

Table 4 illustrates the results of MANOVA examining the effects of gender on CPEP-3 subtest scores for the autistic group. Because there were 13 dependent variables included in the analyses, Bonferroni adjustment was employed to control the experiment-wise error rate, with a family-wise Type 1 error of 0.004. Based on this criterion, the multivariate result was non-significant for gender, F(13, 441) = 2.35, Wilks’ Lambda = 0.94, *p* = .005. As can be seen in Table 4, gender effects were non-significant on all subtests except for cognitive verbal/preverbal (CVP, *p* = .003). In other words, males and females had comparable scores on almost all subtests. These results basically confirm Hypothesis 1c.

**Table 4:** Comparison of males and females within the autistic group (N = 455)

Performance subtests	Female (N = 66)		Male (N = 389)		F value	Sig
	Mean	SD	Mean	SD		
CVP	39.83	20.08	46.93	17.44	8.93	.003
EL	20.32	17.22	24.03	14.82	3.36	.067
RL	21.92	13.74	26.23	11.39	7.55	.006
FM	31.41	7.69	33.64	6.68	6.00	.015
GM	24.77	6.38	27.05	11.42	6.44	.012
VMI	13.67	5.71	14.66	4.82	2.25	.134
AE	15.64	4.49	16.56	4.51	2.37	.125
SR	15.31	5.19	16.16	5.21	1.48	.224
CMB	22.88	6.03	23.84	5.84	1.52	.219
CVB	11.06	7.77	12.72	6.69	3.29	.070
PB	9.83	4.36	9.86	3.60	0.00	.955
PSC	16.61	5.17	17.72	4.37	3.48	.063
AB	18.52	6.04	19.32	5.35	1.24	.266

*CVP* cognitive verbal/preverbal, *EL* expressive language, *RL* receptive language, *FM* fine motor, *GM* gross motor, *VMI* visual-motor imitation, *AE* affective expression, *SR* social reciprocity, *CMB* characteristic motor behaviors, *CVB* characteristic verbal behaviors, *PB* problem behavior, *PSC* personal self-care, *AB* adaptive behavior

### Group Differences (Hypothesis 2)

CPEP-3 Performance subtest scores of normal children were compared with the scores of autistic children. Table 5 shows the means and standard deviations of the two groups and the results of MANOVA. Bonferroni correction was adopted in interpreting the results: given the 10 dependent variables in the analyses, the significant level was adjusted to 0.005 (0.05/11). The multivariate effect of group was significant for the 10 Performance subtest scores as a group, F(11, 724) = 61.30, Wilks’ Lambda = 0.52, *p* < .001. Results of univariate analyses showed significant group differences in all subtests under study, with children in normal group scored significantly higher than children in autistic group using Bonferroni correction (Hypothesis 2a).

**Table 5:** Comparison of the autistic group and the normal group

Performance subtests	Autistic (N = 455)		Normal (N = 281)		F value	Sig
	Mean	SD	Mean	SD		
CVP	45.90	18.01	56.31	10.55	77.44	.000
EL	23.47	15.21	34.54	9.57	119.55	.000
RL	25.64	11.84	34.29	4.33	138.21	.000
FM	33.24	6.86	36.78	3.89	62.30	.000
GM	26.72	10.86	28.58	1.92	49.71	.000
VMI	14.52	4.98	17.97	2.38	118.42	.000
AE	16.43	4.54	20.91	1.62	254.28	.000
SR	16.05	5.20	22.12	2.06	349.57	.000
CMB	23.67	5.89	29.38	1.24	256.14	.000
CVB	12.47	6.85	20.78	1.93	393.88	.000

*CVP* cognitive verbal/preverbal, *EL* expressive language, *RL* receptive language, *FM* fine motor, *GM* gross motor, *VMI* visual-motor imitation, *AE* affective expression, *SR* social reciprocity, *CMB* characteristic motor behaviors, *CVB* characteristic verbal behaviors

### Interrelationships Among Subtests (Hypothesis 3)

Table 6 shows the correlation matrix for the subtests’ raw scores. As different subtests of CPEP-3 measure different aspects of autistic children’s development and behavior, it was hypothesized that these subtests would be moderately correlated with each other. All coefficients were statistically significant (*p* < .01). The coefficients ranged from 0.44 to 0.94, with a mean inter-correlation coefficient of 0.69. These findings indicate that the CPEP-3 subtests measure different aspects of behaviors and developmental skills and provide support for the construct validity of CPEP-3.

**Table 6:** Inter-correlation coefficients of CPEP-3 subtests

Subtests	CVP	EL	RL	FM	GM	VMI	AE	SR	CMB	CVB	PB	PSC
CVP	–	–	–	–	–	–	–	–	–	–	–	–
EL	0.91	–	–	–	–	–	–	–	–	–	–	–
RL	0.94	0.92	–	–	–	–	–	–	–	–	–	–
FM	0.91	0.78	0.85	–	–	–	–	–	–	–	–	–
GM	0.80	0.67	0.78	0.88	–	–	–	–	–	–	–	–
VMI	0.90	0.81	0.88	0.88	0.82	–	–	–	–	–	–	–
AE	0.75	0.74	0.76	0.68	0.59	0.69	–	–	–	–	–	–
SR	0.82	0.82	0.83	0.74	0.68	0.80	0.79	–	–	–	–	–
CMB	0.78	0.73	0.78	0.75	0.66	0.77	0.78	0.79	–	–	–	–
CVB	0.84	0.87	0.86	0.73	0.65	0.78	0.73	0.81	0.75	–	–	–
PB	0.54	0.53	0.54	0.49	0.44	0.51	0.45	0.52	0.51	0.52	–	–
PSC	0.70	0.62	0.64	0.69	0.66	0.66	0.50	0.57	0.52	0.57	0.55	–
AB	0.57	0.53	0.54	0.51	0.46	0.54	0.46	0.51	0.51	0.53	0.71	0.65

The results were based on the autistic sample (N = 455)

*CVP* cognitive verbal/preverbal, *EL* expressive language, *RL* receptive language, *FM* fine motor, *GM* gross motor, *VMI* visual-motor imitation, *AE* affective expression, *SR* social reciprocity, *CMB* characteristic motor behaviors, *CVB* characteristic verbal behaviors, *PB* problem behavior, *PSC* personal self-care, *AB* adaptive behavior

### Factor Structure Based on Confirmatory Factor Analysis (Hypothesis 4)

**Fig. 1:**
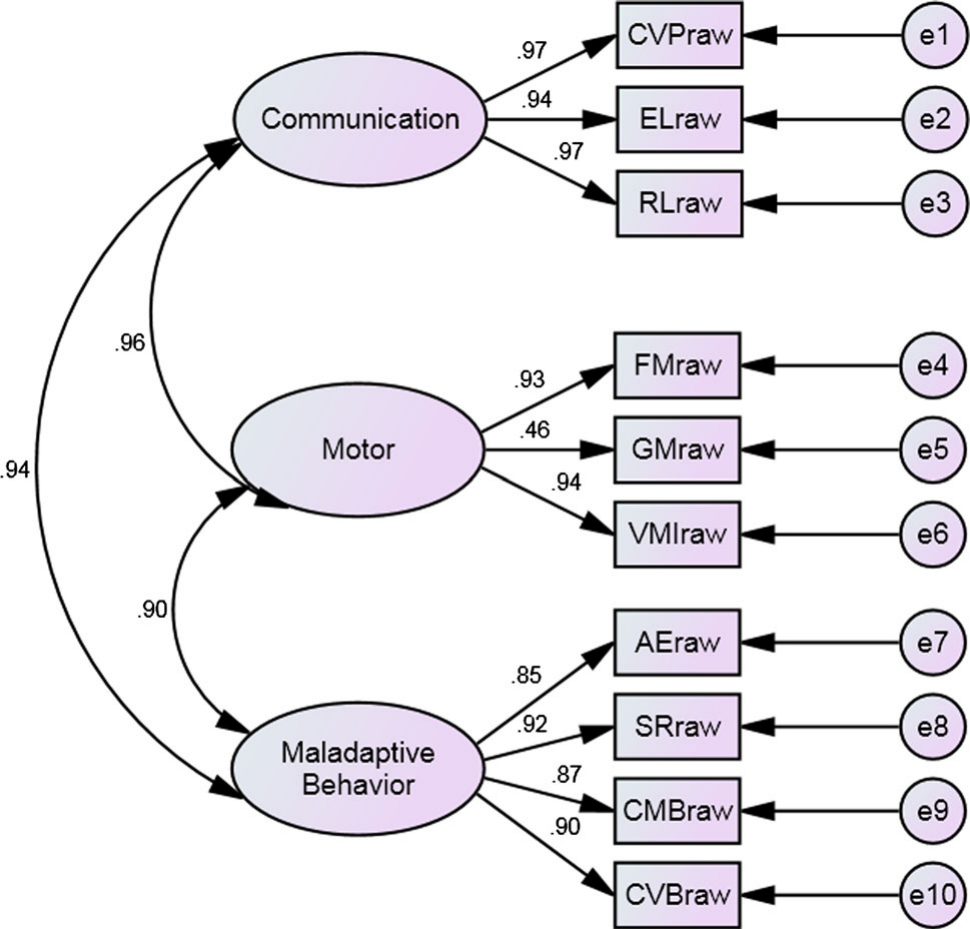
Confirmatory factor analysis of the CPEP-3 illustrating the factor loadings of each subtest of the performance subtests on their respective composite

Results of the confirmatory factor analysis for the three-factor model are presented in Fig. 1. The three composites: communication, motor, and maladaptive behaviors, are represented as ovals. Values on the arrows from the composites to their subtests are factor loadings, representing for the influence of the three factors on their respective subtests. As can be seen in the figure, the sizes of factor loadings are from moderate to very large.

Table 7 summarizes the results of model fit indexes. Almost all indexes supported the fit of the model to the current data, with the CFI equal to 0.944, the TLI equal to 0.921, the NFI equal to 0.939, and the SRMR equal to 0.027. These results are highly comparable to the findings on American samples reported by the PEP-3 developers (Schopler et al. 2005). As presented in Table 8, factor loadings of subtests on their respective factors and correlation coefficients among factors ranged from moderate to high. Based on these findings, it is concluded that the three-factor model is a valid underlying structure that contributes to the ten CPEP-3 Performance subtests.

**Table 7:** Confirmatory factor analysis results

Model Tested	χ^2^	Df	CFI	TLI	NFI	SRMR	RMSEA
CPEP-3 performance	370.69	32	0.944	0.921	0.939	0.027	0.15
Criterion for goodness of fit	–	–	≥0.90	≥0.90	≥0.90	≤0.08	≤0.10

The results were based on the autistic sample (N = 455)

*CFI* comparative fit index, *TLI* Tucker and Lewis’s index of fit, *NFI* normed fit index, *SRMR* standardized root mean square residual, *RMSEA* root mean square error of approximation

**Table 8:** Factor loadings of subtests on their respective factors and correlation coefficients among factors in the three-factor model of CPEP-3

	Communication	Motor	Maladaptive behavior
*Performance subtests*			
CVP	0.97	–	–
EL	0.94	–	–
RL	0.97	–	–
FM	–	0.93	–
GM	–	0.46	–
VMI	–	0.94	–
AE	–	–	0.85
SR	–	–	0.92
CMB	–	–	0.87
CVB	–	–	0.90
*Factors*			
Motor	0.96	–	–
Maladaptive behavior	0.94	0.90	–

The results were based on the autistic sample (N = 455)

### Internal Consistency

The item-total correlation coefficients for each subtest of CPEP-3 were calculated. Table 9 shows the median item-total correlation coefficients for the 13 subtests based on the autistic sample. The values ranged from 0.54 to 0.83, suggesting good internal consistency of the CPEP-3 subtests. The findings are also comparable to previous reports on PEP-3 with Western participants (Schopler et al. 2005).

**Table 9:** Median item-total correlation coefficients for the CPEP-3 subtests

	Overall
*Performance subtests*	
CVP	0.72
EL	0.83
RL	0.80
FM	0.61
GM	0.59
VMI	0.68
AE	0.66
SR	0.63
CMB	0.64
CVB	0.76
*Caregiver report subtests*	
PB	0.54
PSC	0.59
AB	0.55

The results were based on the autistic sample (N = 455)

## Discussion

Children with ASD often display various types of symptoms which make it essential to develop psychometrically sound assessment that can both effectively capture autistic children’s characteristic behaviors and accurately identify their developmental strengths and weaknesses. While it is convenient to translate and adapt well-developed instruments on ASD in different populations, its cross-cultural applicability must be carefully examined. In fact, studies have shown that Chinese translated scales did not show the original dimensions embedded in the original English version (Shek 1998, 2001, 2002). Hence, there is a strong need to validate translated measures in different Chinese contexts.

The present study attempted to examine the construct validity of the Chinese PEP-3. There are several lines of evidence supporting its construct validity. First, consistent with our prediction that children’s cognitive and motor functioning develops with age while maladaptive behaviors would be less related to age (Greenspan and Wieder 1997), significant correlations with age were detected in seven subtests of CPEP-3 that measure developmental skills, including cognitive verbal/preverbal, expressive language, receptive language, fine motor, gross motor, visual-motor imitation, and personal self-care in both the autistic sample and the normal sample, with older children scored higher than younger children in these areas. The findings give support to Hypothesis 1a.

On the other hand, the correlation coefficients between age and six subtests assessing behaviors (i.e., affective expression, social reciprocity, characteristic motor behaviors, characteristic verbal behaviors, problem behavior, and adaptive behavior) were relatively weak among which social reciprocity (r = 0.38) and characteristic verbal behaviors (r = 0.36) had the highest correlations with age. Although a lack of give-and-take of social interaction and appropriate verbal behaviors are typical features of ASD, it is possible that children’s ability in reading social cues and perspectives of others can be improved as they grow older and receive more home-based training from their caregivers (Sheinkopf and Siegel 1998). This may explain the age difference in social reciprocity and characteristic verbal behavior. Generally, these findings provide support to Hypothesis 1b.

Furthermore, consistent with the hypothesis, overall gender differences were non-significant using Bonferroni correction within the autistic children. Further analyses showed that gender differences were non-significant for all subtests except one subtest measuring CVP. These findings basically support the construct validity of CPEP-3 (i.e., Hypothesis 1c). Nevertheless, gender difference found in CVP is an interesting finding which deserves further discussion. Autistic boys showed better performance than did autistic girls in problem solving, verbal naming, sequencing and visual-motor integration, as assessed by CVP. Furthermore, despite the non-significant gender difference, there seems to be a tendency that boys displayed higher level of functioning than did girls in other aspects. Does it mean that autistic boys generally had better developmental level than autistic girls? In fact, similar findings were reported by previous researchers. For example, Wing (1981) found that among people with high-functioning autism, the male to female ratio was about 15:1. On the other hand, in children with low-functioning ASD there were only twice as many boys as girls. This appears to suggest that although girls are less likely to develop ASD, they have more severe problems when they do. Some researchers speculated that boys are more noticeably different or disruptive than girls with the same underlying deficits, whereas girls with high functioning ASD may be better at hiding their difficulties in order to fit in with their peers. As a result, only when girls displayed severe ASD related problems, they are referred for diagnosis, and thus in available statistics girls with ASD seem to be more severely impaired (Attwood 2000; Ehlers and Gillberg 1993; Wing 1981). These may partially explain the gender difference in CVP, while further studies are needed to confirm these hypothesized reasons.

The present study also examined the ability of the CPEP-3 in differentiating children with ASD and their normally developing peers. As predicted, children in the normal group performed better than the autistic group in all 10 subtests they completed. The findings support Hypothesis 2. The findings also echo the results reported by Schopler et al. (2005) on samples of children in the United States and provide evidence for the validity of CPEP-3.

Third, as different subtests of CPEP-3 were designed to measure different developmental and behavioral aspects in children with ASD, it was expected that moderate to large correlations would exist among the subtests. This hypothesis was supported by the present findings (i.e., Hypothesis 3). Fourth, according to the PEP-3 developer (Schopler et al. 2005), the ten subtests of Performance test are theoretically categorized into three composites: communication, motor and maladaptive behaviors. Whether such a three-factor model also applies to Chinese children needs to be tested. The results of confirmatory factor analysis in this study demonstrated a satisfying model fit to the current data based on Chinese children with ASD, suggesting that dividing the Performance test into three dimensions is meaningful when it is used in Chinese population. Hence, the findings supported Hypothesis 4.

Finally, the internal structure of each subtest was found to be homogenous in the present study as reflected in the high item-total correlation coefficients. This indicates that items under each subtest are measuring the general quality that they were designed to measure. Despite the fact that internal consistency is the most widely used measure of reliability, it helps researchers to understand the construct of a scale/subscale by examining the relationships between the item response and total score of the subtest and showing whether the items included in a test/subtest are really complementary and related. In this sense, internal consistency supplements our understanding of validity (Tavakol and Dennick 2011). Altogether, the above findings can be regarded as sound evidence for the construct validity of the CPEP-3.

It is noteworthy that the present study is the first scientific study that investigated the construct validity of the Chinese version of PEP-3 (CPEP-3) on a large sample of children with and without ASD in Hong Kong. In conjunction with the previous validation findings on the reliability, content validity, and concurrent validity of CPEP-3 (Shek and Yu 2013), the present study supports the cross-cultural applicability of this instrument for children with ASD in Chinese contexts.

However, several limitations of this study should be acknowledged. First, while the general sample size was reasonably large, the number of children with age ranging from 7 to 7.9 years was limited (six autistic children and two normal children). This may cause biased and uninterpretable results of the analyses for this age group. While the PEP-3 was developed for children with ASD with a developmental age between 6 months and 7 years, more autistic children above the age of 7 years with low functioning should be included in future studies. Second, the present study was conducted in Hong Kong where the medium of instruction was usually Cantonese. To further generalize the present finding, similar studies must be carried out in other Chinese contexts, including both Mandarin-speaking and Cantonese-speaking communities. Third, while the present study compared children with and without ASD on various CPEP-3 subtests, it would be meaningful to further investigate whether and to what extent the instrument can reflect the developmental differences between high-functioning and low-functioning autistic children. In future study, the severity of ASD for each autistic participant should be rated to make further comparison. Finally, while the factorial validity of the CPEP-3 Performance test was supported by the CFA results, it would be ideal if factorial invariance of the instrument could be examined across different cultural groups, such as autistic children in Hong Kong and in the United States. In this way, knowledge about whether the scores of the instrument could be compared cross-culturally can be accumulated.

Cronbach and Meehl (1955) proposed that construct validity is important for every psychological test, which shall be evaluated by integrating evidence collected from different sources. Although it is impossible for researchers to examine all testable hypotheses related to construct validity, the more strategies used to demonstrate the validity of a test with convincing evidence, the more confidence test users would have in the construct validity of the test. Despite the limitations, the present study can be regarded as a useful contribution for the research and service of autistic children. With reference to four different aspects of construct validity, the present study provided good support for the construct validity of the Chinese version of Psycho-Educational Profile 3rd edition (CPEP-3) by giving a convincing set of validity arguments derived from the results.

There are both theoretical and practical implications of the present study. Theoretically, the findings provide support for the use of CPEP-3 in measuring autistic children in the Chinese context and add to the limited literature on validated instruments for Chinese children with ASD. Practically, this study suggests that the CPEP-3 would serve as a credible and valid measure for professionals to better assess and monitor the development of children with ASD in Hong Kong and other Chinese communities. This would further assist researchers to plan and develop individualized educational programs/projects according to children’s different developmental level.
